# Gait Characteristics of Fallers and Nonfallers in Female Patients with Unilateral End-Stage Hip Osteoarthritis

**DOI:** 10.3390/healthcare13060654

**Published:** 2025-03-17

**Authors:** Yu Kiko, Hirotaka Uchitomi, Masaaki Matsubara, Yoshihiro Miyake

**Affiliations:** 1 Department of Rehabilitation, Tamagawa Hospital, Tokyo 158-0095, Japan; 2 Department of Computer Science, Institute of Science Tokyo, Yokohama 226-8503, Japan; uchitomi@c.titech.ac.jp (H.U.); miyake@c.titech.ac.jp (Y.M.); 3 Department of Orthopedic Surgery, Tamagawa Hospital, Tokyo 158-0095, Japan

**Keywords:** hip osteoarthritis, fallers, gait characteristics, waist trajectory

## Abstract

Background/Objectives: Osteoarthritis of the hip (hip OA) may increase the risk of falls. To decrease fall risk, it is important to assess the gait characteristics of patients with hip OA in detail. This study aimed to investigate the gait characteristics of patients with hip OA caused by falls by simultaneously assessing foot and waist trajectories via an inertial measurement unit (IMU). Methods: The subjects were 77 patients with hip OA, 17 of whom had fallen in the past year. The physical function, gait parameters, and waist trajectories of the fall and nonfall groups were compared. Results: Compared with the nonfall group, the fall group was older and had higher fall scores and lower Japan Orthopaedic Association (JOA) hip scores. With respect to gait characteristics, the stride length on the nonaffected side was significantly shorter in the fall group than in the nonfall group. Stride velocity gait asymmetry was also significantly lower in the fall group than in the nonfall group. The amount of lifting of the waist on the affected and nonaffected sides was significantly lower in the falling group than in the nonfalling group. Conclusions: It was suggested that the fall group might be adapting to stabilization and adjusting to a stable and safe gait pattern because of the effects of falls; new gait characteristics regarding falls in patients with hip OA were found.

## 1. Introduction

Musculoskeletal disorders, including osteoarthritis (OA), are among the most important disabling factors worldwide. Among the 291 conditions worldwide, hip and knee OA is the 11th highest contributor to disability worldwide and the 38th highest contributor to disability [1]. OA is increasing globally, partly due to the aging of the population, and OA in the hip is projected to grow more than OA in the knee between 2020 and 2050 [2]. Patient hip OA is common in females [3] and is a chronic progressive disease characterized by the degeneration and wear of articular cartilage, resulting in joint destruction and reactive bone growth (osteochondria and osteophytes) [4,5]. Limited range of motion (ROM), inguinal pain, and limping are characteristics, and their effects cause activity limitations and quality of life [6,7,8]. Patients with hip OA have a higher risk of death than healthy individuals, and history of diabetes, cancer, or cardiovascular disease and gait disorders are listed as major risk factors [9]. Hip and knee OA can result in reduced foot clearance, difficulty in obstacle avoidance, gait disturbances, and balance issues, thereby increasing the risk of falls [10]. It has also been reported that the risk of falling is increased in elderly women with self-reported lower extremity arthralgia and back pain [10]. Women with fibromyalgia showed reduced ankle dorsiflexion during the stance phase, and a high number of falls was correlated to reduced ankle dorsiflexion during the stance phase [11]. Age-related changes in ankle kinematics and kinetics may be secondarily related to hip flexion contracture impairment rather than impairment at the ankle per se [12]. Patients with end-stage female hip OA had a greater incidence of falls within one year, and fall risk was associated with claudication and knee extension muscle strength [13]. Elderly patients with hip OA have experienced falls or near falls, possibly due to gait disturbances related to hip OA [14]. However, since most falls can occur during forward gait [15], an accurate and detailed assessment of the gait of patients with hip OA is important to prevent falls.

Gait characteristics of patients with hip OA are often reported to be reduced walking speed due to short stride length and short affected limb steps [16,17,18]. The step length [19,20,21] and stance time [17,22] of the affected limb are shortened, resulting in asymmetry in step length and swing time between the affected and unaffected limbs. In end-stage hip OA patients, the lateral distance of the swing phase in patients with hip OA was located significantly more medially on the unaffected side than on the affected one. The lateral distance may indicate joint instability and compensatory gait strategies due to postural control problems [23]. In patients with hip OA, the variability of walking on the treadmill was greater on the affected side than in healthy controls (HCs), but the variability was lowest when the gait speed was close to the self-selected walking speed [24]. On the other hand, total hip arthroplasty (THA) is a treatment for hip OA, but even one year after surgery, gait speed remains slower than that of HCs [25]. Stride and trunk variability are greater in patients with hip OA than in HCs, with improved stride variability but insufficient improvement in trunk variability one year after THA surgery [26]. Patients with hip OA often exhibit exaggerated lateral bending of the pelvis and trunk to the affected side during gait, which is called Duchenne limp [27,28]. Patients with hip OA with Duchenne claudication had a greater thoracic angle during gait [29]. Patients with early hip OA also have greater pelvic movement than HCs [30]. Variability in swing time and stride length has been implicated in predicting fall risk in elderly individuals [31]. In addition, according to the effect size analysis, spatiotemporal parameters such as step length, gait speed, stride length, and stance time variability were respectively more capable of differentiating faller from nonfaller elderlies [32]. However, although there are many reports on spatiotemporal parameters in patients with hip OA, few reports have investigated the associations between spatiotemporal parameters and falls. Therefore, the aim of this study was to investigate the spatiotemporal gait characteristics in patients with hip OA among fallers. In recent years, there has been an increase in gait analysis of OA patients with inertial measurement unit (IMU) sensors, which have provided an accessible and affordable alternative to traditional optical gait analysis systems [33].

This study aims to investigate gait parameters and waist trajectory during walking in unilateral patients with end-stage hip OA who have experienced a fall in the past year via IMU sensors.

## 2. Materials and Methods

### 2.1. Participants

The participants were 82 female patients with end-stage unilateral hip OA (mean age 65.7 ± 7.2 years; 44 with left-sided OA and 38 with right-sided OA) who visited the orthopedic department of Tamagawa Hospital, Nissan Koseikai, from 2022 to 2023. Sample size calculations were performed using **GPower (version 3.1)**. Based on an expected effect size of 0.5 (moderate effect), α = 0.05, and power = 0.80, the minimum sample size required per group was determined to be 64 and the total sample size 128. However, due to the limited availability of fallers, the final sample size was 19 fallers and 63 nonfallers. To assess the impact of this sample size limitation, a post hoc power analysis was performed. We excluded patients if they had neurological, vascular, or other lower extremity musculoskeletal conditions or psychiatric disorders that affected gait or functional performance. We also excluded patients with a self-reported lack of sensation in the foot or lower limb those who were unable to walk short distances (20 m) without an assistive device, and patients with pain in the hip on the unaffected side. Additionally, patients with end-stage deformity or pain in the contralateral hip joint were excluded. This study was approved by the Institutional Review Board of Nissan Tamagawa Hospital and the Tokyo Institute of Technology research ethics committee. All participants provided written informed consent before participation. All methods were performed in accordance with the Declaration of Helsinki and relevant guidelines and regulations.

### 2.2. Fall Assessment

The participants were surveyed via a questionnaire detailing the number and circumstances of falls over the past year [13]. The circumstances of the falls were surveyed: location (indoors, outdoors, stairs), time (morning, daytime, night-time), direction (forward, sideways, backward), cause (slipping, tripping, losing balance, colliding with something), and injury (none, abrasion, contusion, fracture). Falls due to extraordinary environmental factors (e.g., traffic accidents and falls while riding a bicycle) and dizziness were excluded from the count. The Fall Risk Index (FRI-21) is a self-administered questionnaire consisting of 21 items: 8 items related to physical function, 8 items related to disease or geriatric syndromes, and 5 items related to environmental factors; it is often used as a simple fall risk screening method [34].

### 2.3. Assessment of Physical Function

The hip joint ROM angles were determined by passively moving the patients’ legs and measuring the maximum angles via a goniometer. The pelvis was stabilized to prevent rotation or tilting when the passive ROM was measured by an experienced physical therapist [35]. ROM was performed with the patient in the supine position, except for the measurement of the angle of external and internal hip rotation. The external and internal rotation angles were measured in the prone position at 90 knee joint flexion. Knee extension muscle strength was measured while the participants were in a seated position via a handheld dynamometer (μTAS F-1, Anima Co., Ltd., Tokyo, Japan), with the sensor pad fixed to the distal lower leg. The maximum isometric contraction was measured three times on each side for five seconds, with the highest value recorded as the weight ratio (kgf/kg). Hip abduction muscle strength was measured similarly to that of the patient in the supine position and the sensor pad was fixed to the distal thigh. This study employed a standardized measurement protocol [36] and all measurements were performed by experienced physiotherapists to minimize measurement error. Spina malleolar distance (SMD) was measured using a common tape measure to measure the distance between the anterior superior iliac spine and the medial malleolus. The minimum joint space (MJS) was measured via plain X-rays by an orthopedic surgeon. Hip function was assessed via the Japan Orthopaedic Association (JOA) hip score, which includes pain (40 points), ROM (20 points), walking ability (20 points), and activities of daily living (20 points), totaling 100 points. The JOA hip score is designed for the Japanese lifestyle and strongly correlates with the Harris hip score [37]. Hip pain during walking was measured via a visual analog scale (VAS). Before the assessment, all participants received standardized instructions regarding the use of the VAS. The purpose of the VAS was clearly explained, and participants were encouraged to ask questions if they had any uncertainties. The stage of hip OA was classified by an orthopedic surgeon according to the Kellgren–Lawrence classification.

### 2.4. Equipment and Gait Measurement

**Figure 1:**
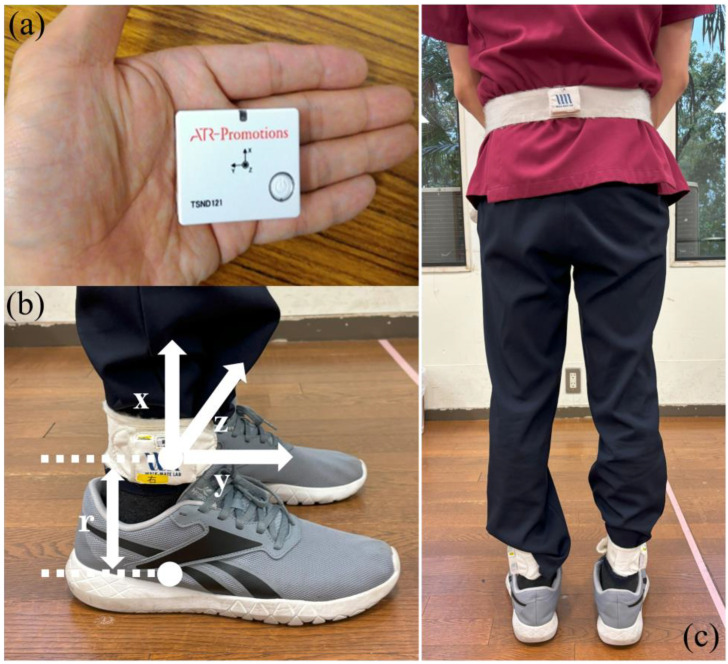
Configuration of the IMUs. (**a**) The IMU with the accelerometer and gyroscope. (**b**) The special band for wearing the IMU on the shank. (**b**) The position and coordination of the IMU. Two IMUs are attached to the shank just above the malleolus at a distance of r. IMUs are attached to the shank in the position 0.03 m above the malleolus. The axes x, y, and z are the coordinate system of the IMU, where the *z*-axis is perpendicular to the sagittal plane formed by the y- and z-axes. (**c**) A third IMU sensor was attached above the L3 lumbar vertebra using a band.

Three IMU sensors (TSND121, ATR-Promotion) were used for gait measurement (Figure 1a). Two sensors were attached above the lateral malleoli using special bands to measure acceleration and angular velocity and estimate gait parameters (Figure 1b). The third IMU sensor was attached above the L3 lumbar vertebra via a band (Figure 1c). The acceleration and angular velocity ranges were ±8 G and ±1000 dps, respectively, with a sampling frequency of 100 Hz. The TSND121 sensor measures 37 mm × 46 mm × 12 mm and weighs approximately 22 g.

**Figure 2:**
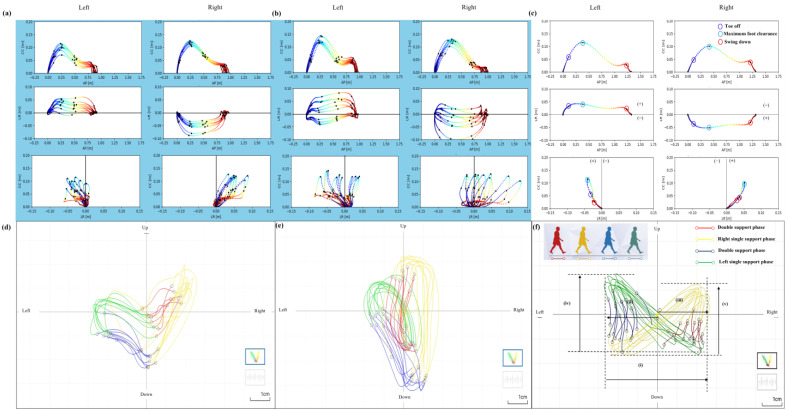
An example for each group of three dimensions estimated of the foot trajectory during walking, obtained from the shank, and an example for each group of a graph of the waist trajectory, visualizing the temporal changes in displacement of the waist during walking. (**a**) An example of the foot trajectory of a patient with left-affected hip osteoarthritis in the fall group. (**b**) An example of the foot trajectory of a patient with left-affected hip osteoarthritis in the nonfall group. (**c**) An example of a foot for the left and right legs. The black dots indicate the positions of toe off, maximum foot clearance, and swing down during the swing phase of walking. In the horizontal and frontal planes, the midline was set as 0, and the lateral distance (LD) from the midline was examined with the swing leg side as (+) and the stance leg side as (−). From left to right and top to bottom: sagittal, horizontal, and frontal planes. Anterior–Posterior: AP; cranio-caudal: CC; left–right: LR. (**d**) An example of the waist trajectory of a patient with left-affected hip osteoarthritis in the fall group. (**e**) An example of the waist trajectory of a patient with left-affected hip osteoarthritis in the nonfall group. (**f**) An example of waist trajectory. Trajectory of the waist during the double support phase—red line; right single leg support phase—yellow line; double support phase—blue line; left single leg support phase—green line. (i) Lateral sway of the lumbar, (ii) lateral sway of the lumbar to the left side, (iii) lateral sway of the lumbar to the right side, (iv) the amount of waist lifting on the left side, (v) the amount of waist lifting on the right side.

The spatiotemporal gait parameters were measured using the WALK-MATE GAIT CHECKER Pro from WALK-MATE LAB Co. Ltd., Tokyo, Japan. For reference, this system performed estimations via the method proposed by Mao et al. [38] and Hori et al. [39]. The method proposed involves the double integration of linear acceleration converted from IMU orientation data. To reduce integration drift errors, a linear error model applied to an inverted pendulum model was introduced to estimate and update velocities during mid-stance. The motion of the IMU during mid-stance can be modeled as a rotational motion in three-dimensional space. The position vectors r and angular velocity in the laboratory coordinate system were calculated from the estimated orientation of the IMU [38]. In this study, short-duration walking tasks were employed, further reducing the potential for drift-related errors. With this method, we estimated the maximum foot clearance, stride length, stride speed, stride duration, stance duration, and swing duration for the left and right lower limbs. The spatiotemporal gait parameters of gait asymmetry were quantified using the gait asymmetry (GA) [40]. GA = 1.0 indicates full symmetry while GA > 1 indicates full asymmetry. We also defined the waist trajectory using a graph visualizing the time evolution of displacement of the waist while walking based on the IMU attached to the waist, as proposed by Kobayashi et al., and calculated the symmetry of lateral sway of the waist trajectory and the amount and symmetry of left and right lifting [41]. The symmetry of the lateral sway of the waist trajectory was calculated with $\frac{\left(ⅱ)-(ⅲ\right)}{\left(ⅱ\right)+(ⅲ)}$  (Figure 2f) and the symmetry of left and right lifting with $\frac{\left(ⅳ)-(ⅴ\right)}{\left(ⅳ\right)+(ⅴ)}$   (Figure 2f) [41].

The participants walked 16 m (10 m with 3 m aids before and after) at a self-selected speed (comfortable), down a straight, flat corridor in comfortable, flat-soled shoes. All participants were measured without any walking aids.

### 2.5. Statistical Analysis

The participants were divided into fallers (those who experienced falls in the past year) and nonfallers. Basic information, FRI-21 score, medical history, physical function of the affected and unaffected sides, GA, walking pain, and waist lateral displacement during walking were assessed for normality via the Shapiro—Wilk test and equal variance by Levene’s test. Comparisons were made via Student’s *t*-test and the Mann—Whitney U test. The means of each spatiotemporal parameter, lateral distances at the swing phase, each standard deviation (SD), and spatiotemporal parameters’ coefficient of variation (CV) were analyzed using two-way mixed ANOVA. The factors for the ANOVA were two groups (fallers and nonfallers) and two legs (affected and unaffected legs). For multiple comparison, we used Shaffer’s method. All statistical analyses were performed using R and the significance level was set at 5%.

## 3. Results

Among the 82 participants, 19 patients with hip OA had fallen at least once in the past year. In addition, 53 patients had experienced a near-miss of a fall in the past year. Furthermore, 32 percent had injuries when they fell, in the following order: fractures (5%), abrasions (12%), and bruises (15%). Among the 19 patients, the most common place of fall was outdoors (52%), followed by indoors (32%) and stairs (16%). Falls occurred during the day (67%), with only a few occurring at night (28%) and in the morning (5%). The most common direction of fall was forward (67%), followed by sideways (29%) and backward (4%). The main causes of falls were tripping (48%), losing balance (33%), and slipping (19%).

Data from 77 of the 82 participants were included in the analysis after exclusion of those with missing values in the questionnaire and gait data. Among these 77 participants, 17 had experienced falls in the past year. A post hoc power analysis was performed to assess the statistical power given the final sample size (n = 17 for fallers, n = 60 for nonfallers). With an effect size of 0.5 (moderate effect), α = 0.05, and a two-tailed independent *t*-test, the statistical power was 0.43 (43%), indicating that the study may have been underpowered to detect moderate effect sizes. To address this limitation, effect sizes are reported alongside *p*-values to facilitate interpretation. In terms of basic information, the faller group was significantly greater in terms of age (t(42) = −2.73, *p* = 0.009, r = 0.3873, 95% CI [−7.044, −1.056]) and FRI-21 score (t(75) = −2.11, *p* = 0.038, r = 0.2366, 95% CI [−2.945, −0.084]), but there were no differences in the other items (Table 1).

There were no significant differences in hip ROM, hip abductor muscle strength, or knee extension muscle strength between the faller and nonfaller groups on the affected and unaffected sides. Similarly, there were no significant differences in the 10-m walking speed or number of steps between the groups (Supplementary Table S1). The overall JOA hip score was significantly lower in the affected (t(75) = 2.61, *p* = 0.011, r = 0.288, 95% CI [2.113, 15.767]) and unaffected (W = 673.5, *p* = 0.044, r = 0.228) groups than in the nonfaller group. In addition, in the JOA hip score subcategories, only the activities of daily living (ADL) score was significantly lower in the faller group than in the nonfaller group (W = 675, *p* = 0.038, r = 0.236) (Table 2).

An example for each group and an HC of the three dimensions estimated of the foot trajectory during walking, obtained from the shank, and an example for each group and an HC of a graph of the waist trajectory visualizing the temporal changes in displacement of the waist during walking are shown in Figure 2a–f.

**Figure 3:**
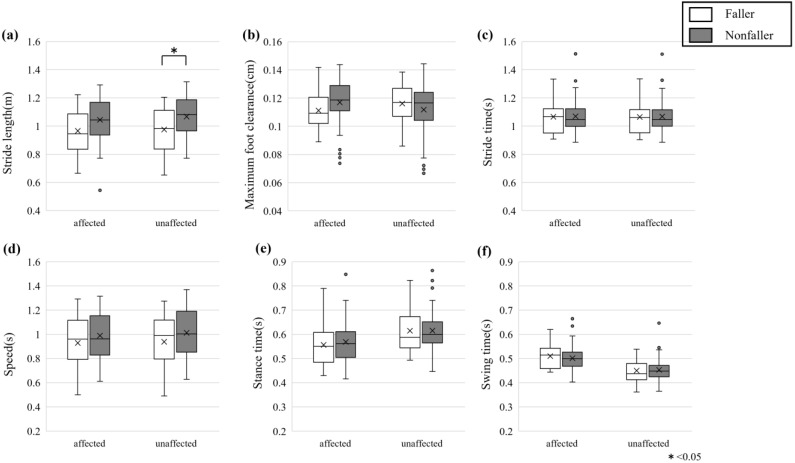
All gait parameters of (**a**) stride length, (**b**) maximum foot clearance, (**c**) stride time, (**d**) speed, (**e**) stance time, and (**f**) swing time. White are the faller groups, gray are the nonfaller groups, and error bars indicate the SD. * indicates significance at *p* < 0.05.

**Figure 4:**
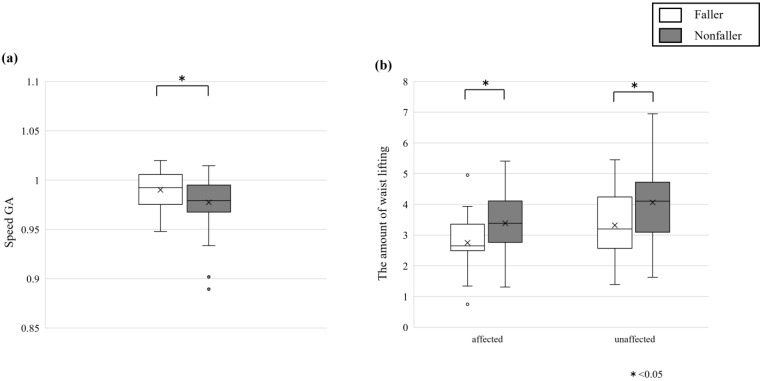
(**a**) Gait asymmetry for speed. (**b**) The amount of waist lifting on the affected side and unaffected side. White are the faller groups, gray are nonfaller groups, and error bars indicate the SD. * indicates significance at *p* < 0.05.

The spatiotemporal gait parameters of the affected and unaffected lower limbs for fallers and nonfallers are shown in Figure 3. Mixed ANOVA was performed for the means of each gait parameter of fallers and nonfallers (Supplementary Table S2). We conducted the post hoc test for these gait parameters. When the simple effect of the group was found, we conducted a multiple comparison using Shaffer’s method. The results are shown in Table 3. One of the differences in spatiotemporal parameters between fallers and nonfallers was that fallers had a significantly shorter stride length (*F* (1, 75) = 3.97, *p* = 0.050, *η*^2^*_p_* = 0.050). At the same time, no significant differences were found for maximum foot clearance, stride time, speed, stance time, or swing time between groups. When comparing affected versus unaffected legs, significant differences were observed in stride length (*F* (1, 75) = 22.21, *p* < 0.001, *η*^2^*_p_* = 0.002), maximum foot clearance (*F* (1, 75) = 4.18, *p* = 0.044, *η*^2^*_p_* = 0.017), speed (*F* (1, 75) = 23.03, *p* < 0.001, *η*^2^*_p_* = 0.001), stance time (*F* (1, 75) = 63.72, *p* < 0.001, *η*^2^*_p_* = 0.060), and swing time (*F* (1, 75) = 68.98, *p* < 0.001, *η*^2^*_p_* = 0.175), with the unaffected side generally showing better performance. A significant interaction effect was found only for speed (*F* (1, 75) = 4.42, *p* = 0.039, *η*^2^*_p_* = 0.001), indicating that the difference in speed between affected and unaffected sides was more pronounced in the faller group. In contrast, no significant interactions were observed for other parameters. Mixed ANOVA was performed for the LD using spatiotemporal gait parameters and the value of each SD (Supplementary Table S3). We conducted the post hoc test for these gait parameters. When the simple effect of the group was found, we conducted a multiple comparison using Shaffer’s method. The results are shown in Supplementary Table S4. The LD at the swing phase of spatiotemporal gait parameters revealed no significant main effects of fallers versus nonfallers between fallers and nonfallers throughout the gait cycle. However, significant main effects in affected versus unaffected individuals were observed in LD at toe off (*F* (1, 75) = 4.36, *p* = 0.040, *η*^2^*_p_* = 0.018), LD at maximum foot clearance (*F* (1, 75) = 10.54, *p* = 0.002, *η*^2^*_p_* = 0.039), and LD at kick out (*F* (1, 75) = 5.83, *p* = 0.018, *η*^2^*_p_* = 0.023), with the unaffected side consistently exhibiting larger lateral positions than the affected side, though no significant difference was found for LD at swing down (*F* (1, 75) = 0.068, *p* = 0.795). No significant interaction effects were observed for LD at the swing phase of spatiotemporal gait parameters. The SD of the spatiotemporal gait parameters revealed no significant main effects for fallers versus nonfallers or affected versus unaffected, nor were there significant interaction effects between fall status and limb for the SD of stride length, max foot clearance, stride time, speed, stance time, LD at toe off, LD at max foot clearance, LD at kick out, and LD at swing down. Regarding the SD of swing time, there was a significant main effect of limb (*F* (1, 75) = 6.01, *p* = 0.017, *η*^2^*_p_* = 0.037), but no significant main effect for fallers versus nonfallers or an interaction effect. Mixed ANOVA was performed for the CV of the spatiotemporal gait parameters (Supplementary Table S5). We conducted the post hoc test for these gait parameters. When the simple effect of the group was found, we conducted a multiple comparison using Shaffer’s method. The results are shown in Supplementary Table S6. The CV of the main effects of stance time for affected versus unaffected was significantly greater on the affected side than on the unaffected side (*F* (1, 75) = 8.507, *p* = 0.005, *η*^2^ = 0.0330), but for other indices, there were no significant main effects, nor any interaction effects. In terms of symmetry indices, GA for speed was significantly lower in the faller group than in the nonfaller group (*p* = 0.049, r = 0.223) (Figure 4a), but there were no significant differences in the other indices (Table 4).

There were no significant differences between the faller and nonfaller groups in terms of the magnitude of lateral sway, asymmetry of lateral sway, or asymmetry in the amount of lifting in the hip orbit (Table 5). The amount of waist lifting on the affected side was significantly lower in the faller group than in the nonfaller group (*p* = 0.019, r = 0.266). The amount of waist lifting on the nonfaller side was also significantly lower in the faller group than in the nonfaller group (*p* = 0.025, r = 0.257) (Figure 4b).

## 4. Discussion

In this study, we compared the spatiotemporal parameters of gait and waist trajectory during walking using IMU sensors in patients with unilateral end-stage hip OA divided into those with and without a history of falls in the past year. The faller groups were older and had higher total FRI-21 scores than the nonfaller groups. A previous study reported an age difference of 2 years between fallers and nonfallers [42], which is consistent with the 4-year difference in the present study. Muscle strength and balance ability decline with age, which may contribute to the risk of falls. Therefore, it is possible that this is due to age-related functional decline rather than age per se [43]. The FRI-21 is a simple questionnaire index and may be able to discriminate past falls. The overall JOA hip score was lower in the faller groups than in the nonfaller groups on both the affected and nonaffected side. In the lower categories, the faller groups were significantly lower in activities of daily living (sitting, standing, squatting and standing up, climbing stairs, getting in and out of cars and buses, etc.). Although there is an association between subjective difficulty in stair climbing and physical functions such as walking speed and muscle strength in elderly individuals [44], there were no differences in walking speed or muscle strength in this study. This discrepancy suggests that hip dysplasia patients may maintain their walking speed by performing compensatory gait adaptations despite difficulties when ascending and descending stairs. Subjective difficulty in stair climbing may also reflect not only muscle strength and walking speed but also psychological factors such as previous falls and fear of falling [44].

The center of gravity shift and ability to shift the center of gravity and postural correction were associated with a fear of falling, and it is possible that the fear of falling increased in the fall group due to previous falls, which may have affected the JOA hip score in activities of daily living. We compared the spatiotemporal parameters of gait and waist trajectory, and the results suggest that patients with a history of falls have a significantly shorter stride length than those without a history of falls. Patients with a history of falls presented significantly less asymmetry in gait velocity than those without a history of falls. It was also suggested that patients with a history of falls had significantly smaller waist lift heights than patients without a history of falls. These are the first results revealed by the simultaneous comparison of spatiotemporal parameters of gait and waist trajectory during walking in this study. A more detailed discussion follows.

Gait parameters were significantly shorter on the nonaffected side in the faller groups than in the nonfaller groups, and stride length tended to decrease on the affected side. The falling group of healthy older adults had lower walking speed, longer stance time, and lower stride length and step width, as well as greater variability in spatiotemporal parameters [32]. In addition, healthy older adults may shorten their stride length to maintain dynamic balance and stability to prevent falls [45], and patients with osteoarthritis may follow a similar strategy. However, we believe that there was no difference in the other parameters between the faller and nonfaller groups because a decrease in velocity or prolonged stance time in patients with hip OA may lead to an exacerbation of pain during loading. The reduced range of motion of the hip increases pelvic movement, resulting in a shorter stride length, while the stance time remains the same to allow time to maintain balance [30]. In addition, there were no differences in the standard deviation values or coefficients of variation of gait parameters between the faller and nonfaller groups. Although there are many reports on the relationship between falls and variability in healthy elderly patients [32], it is possible that patients with hip OA may pattern their gait more than healthy patients do because of pain and a limited range of motion. Therefore, even after THA surgery, improvements in preoperative gait parameters and gait may take longer [25]. Some of the results basically supported previous studies. The results of stride length, speed, stance time, and swing time for affected versus unaffected individuals were consistent with the gait characteristics of individuals with hip OA in a previous study [46]. In a previous report, a group of patients with hip OA experienced a decrease in walking speed [13]. This difference between that study and ours is that patients with various gait levels were included in our study because we did not limit the use of walking aids during indoor walking and gait parameter measurements.

In terms of gait parameter symmetry, the faller groups were significantly lower than the nonfaller groups in terms of speed GA. This was indicated by a greater symmetry of speed in the faller groups than in the nonfaller groups. It has been reported that lower gait symmetry is associated with a greater risk of falls [47]. In this study, falls were retrospectively analyzed via questionnaires. The fall evaluation in this retrospective study was preceded by a fall at the time of evaluation and may have been adjusted to a conservative, stable, and safe gait and movement pattern at the time of measurement [48], and we believe that the fall group in this study also adjusted to a stable gait pattern, resulting in a high degree of symmetry.

The amount of waist lift was lower in the faller groups than in the nonfaller groups on both the affected and nonaffected side. Compared with HCs, hip OA patients have a reduced hip flexion angle while walking [49]. The main cause of falls in this study was also tripping, and decreased minimum leg clearance has been reported to be associated with falls [50]. It is possible that the nonfaller groups prevented falls by compensating for the decreased hip flexion angle and foot clearance with their hips. In addition, the magnitude of the acceleration of the head and pelvis during walking in younger and older adults was smaller than that in older adults, which may be a compensatory strategy to maintain balance [51].

Falls can easily lead to fractures and other injuries, which can rapidly decrease quality of life. Fear of falling can also lead to decreased spatiotemporal parameters [52], decreased physical function and range of activity, and decreased quality of life [53]. Although there was no difference in muscle strength between the faller and nonfaller groups in this study, more than 60% of the participants experienced a near-miss, suggesting that in addition to activity limitations due to hip OA, the fear of falling may have reduced activity. Therefore, interventions to reduce the fear of falling are also necessary to prevent falls.

Exercise instruction has been reported to be effective in preventing falls and reducing fear of falling. Using the gait characteristics identified in this study, we will investigate teaching and intervention methods such as stride length optimization and postural control strategies during hip raising. We also aim to introduce the IMU into clinical settings to enable real-time monitoring of gait, detect early gait abnormalities, and improve fall prevention, fear of falling, and gait by providing visual feedback on gait asymmetry and leg and waist trajectories.

### Limitations of This Study

One limitation of this study is the small sample size in the faller group (n = 17), which may have resulted in insufficient statistical power. The post hoc power analysis revealed that with an effect size of 0.5, the power was only 0.43 (43%), suggesting that this study was underpowered to detect moderate effect sizes. Future studies should consider increasing the sample size to improve statistical power and confirm these findings.

In gait analysis with IMU sensors, there is the potential influence of clothing on the positioning and stability of the IMU sensors. To minimize this effect of this study, special bands were used to secure the sensors in place. However, despite these precautions, minor movements of the sensors due to skin displacement remain a potential source of error. Future studies should consider alternative sensor attachment methods and custom-designed wearable solutions to further reduce motion artifacts.

The association with falls cannot be determined unequivocally because of the retrospective nature of studies on falls. The reproducibility of the gait parameters is reduced because of the arbitrary gait speed and the number of measurements. The location of gait measurement is limited and may differ from that of daily walking.

## 5. Conclusions

This study found that the patients in the fall group were older and reported lower hip function in the questionnaire than nonfall patients. Gait parameters showed reduced stride length in the nonaffected limb and greater GA in terms of stride speed asymmetry in the fall group. These findings may indicate that the falling group may be better adapted to stabilization and adjust to a stable and safe gait pattern after a fall. In terms of waist trajectory, the fall group had lower waist lifting on both the affected and nonaffected side than the nonfall group. It is possible that this may be a strategy to maintain balance due to postfall, but it is also possible that the nonfall group may compensate at the waist to prevent falls. In this study, new gait characteristics were discovered via detailed evaluation of the gait parameters of the falling and nonfalling groups of patients with unilateral end-stage hip OA. In the future, these gait characteristics should be used to determine the relationship between fall risk and interventions for fall prevention.

## Figures and Tables

**Table 1:** Participant demographics.

Variables	Fallers (n = 17)	Nonfallers (n = 60)	*p* Value	95% CI	r
Age (years)	69.0 ± 4.6	65.0 ± 7.5	0.009	−7.044–−1.056	0.387
Stature (cm)	153.8 ± 3.2	155.3 ± 6.0	0.202	−0.783–3.612	0.180
Body mass (kg)	52.7 ± 8.7	54.3 ± 9.1	0.510	−3.303–6.589	0.076
BMI (kg/m^2^)	20.9 (19.5, 24.6)	22.1 (19.7, 24.6)	0.759		0.034
History of present illness (years)	5.2 (2, 10)	6.0 (2, 10)	0.951		0.006
Fall Risk Index	9.8 ± 2.6	8.3 ± 2.6	0.038	−2.945–−0.084	0.237

Mean ± SD, median (interquartile range).

**Table 2:** Comparison of physical function between fallers and nonfallers in patients with hip OA. The JOA hip score and MJS and SMD depict the Japanese Orthopaedic Association hip score and the minimum joint space and spina malleolar distance.

	Fallers (n = 17)	Nonfallers (n = 60)	*p* Value	95% CI	r
JOA hip score total					
Affected	52.2 ± 12.5	61.1 ± 12.5	0.011	2.113–15.767	0.288
Unaffected	81.8 (75, 89)	86.8 (83, 93)	0.044		0.228
JOA hip score pain					
Affected	13.5 (10, 20)	17.8 (10, 20)	0.140		0.168
Unaffected	38.5 (35, 40)	37.9 (35, 40)	0.944		0.007
JOA hip score gait ability	13.9 (10, 18)	14.6 (15, 15)	0.856		0.020
JOA hip score ADL	14.9 (12, 18)	16.8 (16, 20)	0.038		0.236
MJS (mm)					
Affected	0	0			
Unaffected	3.3 ± 1.3	2.8 ± 1.0	0.085	−1.096–0.073	0.199
SMD (mm)					
Unaffected/affected	5.0 (0, 1.0)	0 (0, 1.0)	0.294		0.119

Mean ± SD, median (interquartile range).

**Table 3:** The results of multiple comparison tests for the groups.

Gait Parameters	Affected/ Unaffected	Mean (SD)		*p*-Value	*η* ^2^ *_p_*
		Fallers (n = 17)	Nonfallers (n = 60)	Fallers vs. Nonfallers	
Stride length (m)	Affected	0.97 (0.16)	1.04 (0.15)	0.069	0.043
	Unaffected	0.98 (0.17)	1.07 (0.15)	0.037	0.057
Maximum foot clearance (m)	Affected	0.11 (0.01)	0.11 (0.02)	no significant simple effect	
	Unaffected	0.12 (0.01)	0.12 (0.02)	no significant simple effect	
Stride time (s)	Affected	1.07 (0.13)	1.07 (0.11)	no significant simple effect	
	Unaffected	1.06 (0.13)	1.07 (0.11)	no significant simple effect	
Speed (s)	Affected	0.93 (0.22)	0.99 (0.19)	0.260	0.017
	Unaffected	0.94 (0.22)	1.01 (0.19)	0.173	0.024
Stance time (s)	Affected	0.56 (0.10)	0.57 (0.08)	no significant simple effect	
	Unaffected	0.61 (0.10)	0.61 (0.08)	no significant simple effect	
Swing time (s)	Affected	0.51 (0.05)	0.50 (0.05)	no significant simple effect	
	Unaffected	0.45 (0.05)	0.45 (0.05)	no significant simple effect	

**Table 4:** Comparison of asymmetry in gait parameters between faller and nonfaller patients with hip OA. Lateral distance (LD).

Gait Asymmetry	Fallers (n = 17)	Nonfallers (n = 60)	*p* Value	95% CI	r
Stride length GA	0.99 (0.97, 1.01)	0.98 (0.97, 0.99)	0.116		0.178
Maximum foot clearance GA	0.98 (0.85, 1.06)	0.98 (0.88, 1.03)	1.000		−0.001
Stride time GA	1.00 ± 0.01	1.00 ± 0.00	0.809	−0.004–0.005	0.056
Speed GA	0.99 (0.98, 1.00)	0.98 (0.97, 0.99)	0.049		0.223
Stance time GA	0.91 ± 0.08	0.93 ± 0.07	0.322	−0.020–0.061	0.114
Swing time GA	1.14 ± 0.12	1.11 ± 0.11	0.301	−0.092–0.029	0.120
LD at toe off GA	0.67 (0.34, 0.92)	0.69 (0.27, 1.41)	0.632		0.053
LD at maximum foot clearance GA	0.57 (0.33, 0.95)	0.56 (0.19, 1.32)	0.722		0.040
LD at kick out GA	0.53 (0.28, 1.21)	0.77 (0.22, 1.41)	0.921		0.011
LD at swing down GA	0.38 (−0.49, 0.85)	0.75 (0.17, 1.54)	0.067		0.208

Mean ± SD, median (interquartile range).

**Table 5:** Comparison of waist trajectory between fallers and nonfallers in patients with hip OA.

Waist Trajectory	Fallers (n = 17)	Nonfallers (n = 60)	*p* Value	95% CI	r
Lateral sway of the lumbar	3.16 (2.90, 4.31)	3.52 (2.78, 4.62)	0.425		0.090
Asymmetry in lateral sway of the lumbar	10.23 (4.76, 20.56)	13.58 (4.98, 22.49)	0.469		0.082
Asymmetry in the amount of waist lifting	20.42 (7.39, 41.78)	23.87 (11.33, 44.09)	0.876		0.017
The amount of waist lifting:					
Affected	2.75 ± 1.01	3.39 ± 0.95	0.019	0.106–1.163	0.266
Unaffected	3.32 ± 1.07	4.06 ± 1.22	0.025	0.095–1.399	0.257

Mean ± SD, median (interquartile range).

## Data Availability

The data that support the findings of this study are available from the corresponding author upon reasonable request.

## Supplementary Materials

The following supporting information can be downloaded at https://www.mdpi.com/article/10.3390/healthcare13060654/s1: Supplementary Table S1: Comparison of physical function between faller and nonfaller patients with hip OA. Supplementary Table S2: Mixed ANOVA table of spatiotemporal gait parameters. Supplementary Table S3: Mixed ANOVA table of LD at spatiotemporal gait parameters and the value of each SD. LD and SD depict lateral distance and standard deviation. Supplementary Table S4: The results of multiple comparison tests for the group for LD at spatiotemporal gait parameters and the value of each SD. LD and SD depict lateral distance and standard deviation. Supplementary Table S5: Mixed ANOVA table of CV of the spatiotemporal gait parameters. CV depicts coefficient of variation. Supplementary Table S6: The results of multiple comparison tests for the group for CV of the spatiotemporal gait parameters. CV depicts coefficient of variation.
