# Supplementary material for: Gait Characteristics of Fallers and Nonfallers in Female Patients with Unilateral End-Stage Hip Osteoarthritis
Source: Healthcare (Basel). 2025 Mar 17;13(6):654. doi: 10.3390/healthcare13060654 (PMC11942401; doi:10.3390/healthcare13060654)
Supplement: Supplementary file 1 [file healthcare-13-00654-s001.zip › healthcare-3410333-supplementary.pdf]

Gait Characteristics of Fallers and Nonfallers in Female Patients with Unilateral End-Stage Hip Osteoarthritis

Statistical results

Supplementary Table S1. Comparison of physical function between faller and non-faller of patients with hip OA.

	**Fallers(n＝17)**	**Non-fallers (n＝60)**	**p-value**	**95%CI**	**r**
Flexion(degree)	87.6±15.9	90.0±16.0	0.593	-6.383 11.089	0.062
Affected	107.4(100,115)	106.5(100,115)	0.779		0.031
Unaffected					
Extension(degree)	5.6(5,10)	6.2(5,10)	0.514		0.074
Affected	13.2(10,15)	12.0(10,15)	0.356		0.104
Unaffected					
Abduction(degree)	15.9±6.2	19.3±9.5	0.160	-1.397 8.299	0.162
Affected	28.8(25,35)	30.3(25,35)	0.390		0.097
Unaffected					
Adduction(degree)	10.3(10,10)	9.9(5,10)	0.679		0.046
Affected	15.9(15,20)	14.9(10,16)	0.364		0.103
Unaffected					
External	19.1±10.8	23.4±13.3	0.225	-2.706 11.304	0.140
rotation(degree)	32.1(30,40)	32.5(25,40)	0.828		0.024
Affected					
Unaffected					
Internal	19.1±15.9	16.9±17.1	0.635	-11.407	0.055
rotation(degree)	37.1±13.4	37.3±14.3	0.961	7.005	0.006
Affected				-7.517 7.899	
Unaffected					

Gait pain VAS(mm)	40.9±22.1	32.9±23.2	0.217	-20.632<br>4.758	0.147
10mgait speed(s)	11.1(8.7,11.9)	10.2(8.5,11.1)	0.341		0.108
10mgait steps	19.8（17,22）	19.7（17,21）	0.465		0.083
Knee extension muscle strength(kgf/kg)<br>Affected<br>Unaffected	0.28±0.11<br>0.33±0.13	0.24±0.11<br>0.32±0.13	0.218<br>0.699	-0.098 0.023<br>-0.083 0.056	0.142<br>0.045
Hip abduction muscle strength(kgf/kg)<br>Affected<br>Unaffected	0.15(0.11,0.18)<br>0.21(0.16,0.30)	0.15(0.10,0.18)<br>0.18(0.13,0.21)	0.922<br>0.173		0.011<br>0.155

mean±SD, median(interquartile range)

Supplementary Table S2. Spatiotemporal gait parameters mixed ANOVA table.

**Gait parameters**	**Condition**	***df***	***F-value***	***p-value***	η^2_p
Stride length	Group	1,75	3.97	0.050	0.178
	Affected and unaffected	1,75	22.21	<0.001	0.002
	Group × affected and unaffected	1,75	3.58	0.063	0.001
Maximum foot clearance	Group	1,75	0.04	0.839	0.001
	Affected and unaffected	1,75	4.18	0.044	0.017
	Group × affected and unaffected	1,75	0.00	0.959	0.001

Stride time	Group	1,75	0.01	0.912	0.001
	Affected and unaffected	1,75	2.64	0.108	0.001
	Group × affected and unaffected	1,75	0.02	0.888	0.001
Speed	Group	1,75	1.59	0.212	0.021
	Affected and unaffected	1,75	23.03	0.000	0.001
	Group × affected and unaffected	1,75	4.42	0.039	0.001
Stance time	Group	1,75	0.07	0.788	0.001
	Affected and unaffected	1,75	63.72	0.000	0.060
	Group × affected and unaffected	1,75	0.86	0.356	0.001
Swing time	Group	1,75	0.05	0.821	0.001
	Affected and unaffected	1,75	68.98	0.000	0.175
	Group × affected and unaffected	1,75	0.85	0.359	0.003

Supplementary Table S3. LD at spatiotemporal gait parameters and the value of each SD mixed ANOVA table. LD and SD depicts lateral distance and standard deviation.

	Condition	*df*	*F-value*	*p-value*	η^2_p
LD at toe off	Group	1,75	0.14	0.705	0.001
	Affected and unaffected	1,75	4.36	0.040	0.018
	Group × affected and unaffected	1,75	0.62	0.435	0.002
LD at maximum foot clearance	Group	1,75	0.02	0.886	0.001
	Affected and unaffected	1,75	10.54	0.002	0.039
	Group × affected and unaffected	1,75	0.68	0.412	0.003
LD at kick out	Group	1,75	0.60	0.442	0.006
	Affected and unaffected	1,75	5.83	0.018	0.023
	Group × affected and unaffected	1,75	0.23	0.631	0.001
LD at swing down	Group	1,75	0.72	0.397	0.007
	Affected and unaffected	1,75	0.07	0.795	0.001
	Group × affected and unaffected	1,75	0.25	0.618	0.001
SD of stride length	Group	1,75	0.01	0.935	0.001
	Affected and unaffected	1,75	0.67	0.415	0.002
	Group × affected and unaffected	1,75	2.59	0.112	0.008
SD of maximum foot clearance	Group	1,75	0.88	0.351	0.007
	Affected and unaffected	1,75	0.14	0.705	0.001
	Group × affected and unaffected	1,75	1.21	0.275	0.006
SD of stride time	Group	1,75	1.86	0.177	0.016
	Affected and unaffected	1,75	1.98	0.164	0.009
	Group × affected and unaffected	1,75	0.03	0.858	0.001
SD of speed	Group	1,75	0.01	0.954	0.000
	Affected and unaffected	1,75	1.21	0.275	0.004

	Group × affected and unaffected	1,75	1.46	0.231	0.005
SD of stance time	Group	1,75	2.41	0.125	0.024
	Affected and unaffected	1,75	2.49	0.119	0.007
	Group × affected and unaffected	1,75	0.01	0.934	0.000
SD of swing time	Group	1,75	1.08	0.303	0.008
	Affected and unaffected	1,75	6.01	0.017	0.037
	Group × affected and unaffected	1,75	0.61	0.438	0.004
SD of LD at toe off	Group	1,75	0.83	0.366	0.007
	Affected and unaffected	1,75	3.13	0.081	0.017
	Group × affected and unaffected	1,75	0.23	0.632	0.001
SD of at LD maximum foot clearance	Group	1,75	1.11	0.295	0.008
	Affected and unaffected	1,75	0.81	0.371	0.005
	Group × affected and unaffected	1,75	0.16	0.691	0.001
SD of at LD kick out	Group	1,75	1.34	0.250	0.010
	Affected and unaffected	1,75	1.08	0.302	0.006
	Group × affected and unaffected	1,75	0.09	0.766	0.001
SD of at swing down	Group	1,75	1.24	0.269	0.009
	Affected and unaffected	1,75	0.48	0.492	0.003
	Group × affected and unaffected	1,75	0.09	0.766	0.001

Supplementary Table S4. LD at spatiotemporal gait parameters and the value of each SD the result of multiple comparison tests for the group. LD and SD depicts lateral distance and standard deviation.

	**Affected/ unaffected**	**Mean(SD)**		***p-value***
		Fallers (n＝17)	Nonfallers (n＝60)	Fallers vs Nonfallers
LD at toe off (m)	Affected	0.01 (0.01)	0.02 (0.02)	no significant simple effect
	Unaffected	0.02 (0.01)	0.02 (0.02)	no significant simple effect
LD at maximum foot clearance (m)	Affected	0.02 (0.02)	0.02 (0.03)	no significant simple effect
	unaffected	0.03 (0.02)	0.03 (0.03)	no significant simple effect
LD at kick out (m)	Affected	0.01 (0.02)	0.02 (0.03)	no significant simple effect
	unaffected	0.03 (0.02)	0.03 (0.03)	no significant simple effect
LD at swing down (m)	Affected	0.01 (0.01)	0.01 (0.02)	no significant simple effect
	unaffected	0.01 (0.01)	0.01 (0.02)	no significant simple effect
SD of stride length	Affected	0.04 (0.02)	0.03 (0.02)	no significant simple effect
	unaffected	0.03 (0.01)	0.04 (0.02)	

				no significant simple effect
SD of maximum foot clearance	Affected	0.01 (0.01)	0.01 (0.01)	no significant simple effect
	unaffected	0.01 (0.01)	0.01 (0.01)	no significant simple effect
SD of stride time	Affected	0.04 (0.02)	0.03 (0.02)	no significant simple effect
	unaffected	0.04 (0.02)	0.03 (0.01)	no significant simple effect
SD of speed	Affected	0.05 (0.03)	0.05 (0.02)	no significant simple effect
	unaffected	0.04 (0.02)	0.05 (0.02)	no significant simple effect
SD of stance time	Affected	0.03 (0.03)	0.03 (0.01)	no significant simple effect
	unaffected	0.03 (0.02)	0.03 (0.01)	no significant simple effect
SD of swing time	Affected	0.03 (0.01)	0.02 (0.01)	no significant simple effect
	unaffected	0.02 (0.01)	0.02 (0.01)	no significant simple effect
SD of LD at toe off	Affected	0.01 (0.01)	0.02 (0.02)	no significant simple effect
	unaffected	0.02 (0.01)	0.02 (0.01)	no significant

				simple effect
SD of at LD maximum foot clearance	Affected<br>unaffected	0.02 (0.01)<br>0.03 (0.01)	0.03 (0.03)<br>0.03 (0.02)	no significant simple effect<br>no significant simple effect
SD of at LD kick out	Affected<br>unaffected	0.02 (0.01)<br>0.03 (0.01)	0.03 (0.03)<br>0.03 (0.01)	no significant simple effect<br>no significant simple effect
SD of at swing down	Affected<br>unaffected	0.01 (0.01)<br>0.02 (0.01)	0.02 (0.03)<br>0.02 (0.01)	no significant simple effect<br>no significant simple effect

Supplementary Table S5. CV of the spatiotemporal gait parameters mixed ANOVA table. CV depicts coefficient of variation.

	**Condition**	***df***	***F-value***	***p-value***	η^2_p
CV of stride length	Group	1,75	0.40	0.527	0.004
	Affected and unaffected	1,75	1.10	0.298	0.003
	Group × affected and unaffected	1,75	2.29	0.134	0.007

CV of maximum foot clearance	Group	1,75	0.91	0.342	0.008
	Affected and unaffected	1,75	0.39	0.534	0.002
	Group × affected and unaffected	1,75	1.02	0.317	0.005
CV of stride time	Group	1,75	2.10	0.152	0.016
	Affected and unaffected	1,75	1.81	0.183	0.010
	Group × affected and unaffected	1,75	0.07	0.787	0.001
CV of speed	Group	1,75	0.37	0.545	0.003
	Affected and unaffected	1,75	1.16	0.285	0.005
	Group × affected and unaffected	1,75	0.62	0.434	0.003
CV of stance time	Group	1,75	2.67	0.106	0.024
	Affected and unaffected	1,75	8.51	0.005	0.033
	Group × affected and unaffected	1,75	0.18	0.671	0.001
CV of swing time	Group	1,75	0.78	0.381	0.005
	Affected and unaffected	1,75	0.87	0.356	0.006
	Group × affected and unaffected	1,75	0.26	0.613	0.002

Supplementary Table S6. CV of the spatiotemporal gait parameters the result of multiple comparison tests for the group. CV depicts coefficient of variation.

**Coefficient of Varidation**	**Affected/ unaffected**	**Mean(SD)**		***p-value***
		Fallers(n＝17)	Nonfallers (n＝60)	Fallers vs Nonfallers
CV of stride length	Affected	4.02 (2.24)	3.39 (1.77)	no significant simple effect
	Unaffected	3.43 (1.28)	3.50 (1.82)	no significant simple effect
CV of maximum foot clearance	Affected	6.78 (4.99)	7.01 (7.17)	no significant simple effect
	unaffected	4.94 (2.93)	7.44 (7.29)	no significant simple effect
CV of stride time	Affected	3.69 (1.56)	3.23 (1.39)	no significant simple effect
	unaffected	3.32 (1.45)	2.99 (1.07)	no significant simple effect
CV of speed	Affected	5.30 (2.52)	4.77 (1.98)	no significant simple effect
	unaffected	4.72 (1.84)	4.68 (2.00)	no significant simple effect
CV of stance time	Affected	6.23 (4.02)	5.20 (1.95)	no significant simple effect
	unaffected	5.06 (2.72)	4.33 (1.89)	no significant

				simple effect
CV of swing time	Affected<br>unaffected	5.50 (2.11)<br>4.92 (2.96)	4.93 (2.05)<br>4.76 (1.81)	no significant simple effect<br>no significant simple effect
